# The development of a policy brief on physical activity and health in Africa for children and adolescents with disabilities: COVID-19 and beyond

**DOI:** 10.4102/ajod.v11i0.1100

**Published:** 2022-12-15

**Authors:** Rowena Naidoo, Verusia Chetty, Marie E.M. Young, Phindile E. Mahlalela, Philippe J. Gradidge, Soraya Maart, Dané Coetzee, Brett Smith, Estelle Lambert

**Affiliations:** 1 Discipline of Biokinetics, Exercise and Leisure Sciences, College of Health Sciences, University of KwaZulu-Natal, Durban, South Africa; 2 Discipline of Physiotherapy, College of Health Sciences, University of KwaZulu-Natal, Durban, South Africa; 3 Department of Sport, Recreation and Exercise Science, Faculty of Community and Health Sciences, University of the Western Cape, Cape Town, South Africa; 4 Department of Science and Innovation, Human Sciences Research Council, Pretoria, South Africa; 5 Centre for Exercise Science and Sports Medicine, Faculty of Health Sciences, University of the Witwatersrand, Johannesburg, South Africa; 6 Department of Health and Rehabilitation Sciences, Faculty of Health Sciences, University of Cape Town, Cape Town, South Africa; 7 Department of Physical Activity, Sport and Recreation (PhASRec) Focus Area, Faculty of Health Sciences, North-West University, Potchefstroom, South Africa; 8 Department of Sport and Exercise Sciences, Durham University, Durham, United Kingdom; 9 Department of Human Biology, Faculty of Health Sciences, University of Cape Town, Cape Town, South Africa

## Introduction

The coronavirus disease 2019 (COVID-19) was declared as a global pandemic by the World Health Organization (WHO) in March 2020. To curtail the spread of the virus, governments implemented national lockdowns, restricting the movement of individuals. As the pandemic evolved and vaccine roll-out was implemented the lockdown measures were eased. However, even with the lowering of lockdown measures in South Africa, access to public places was limited: beaches, gyms and recreational facilities were inaccessible to the public. This resulted in an immediate and general reduction in physical activity and participation in sport, both social and competitive, and a subsequent increase in anxiety and depression (Taquet, Holmes & Harrison 2021).

Yet again, children and adolescents with disabilities were particularly vulnerable, as the already limited spaces where they had participated in physical activity were often not universally accessible. The benefits of physical activity for these young people are well known, and include an improvement in cardiovascular fitness, psychosocial and physical functioning and rehabilitation outcomes (Kim et al. 2016). Therefore, as part of a series of policy briefs to promote physical activity for health, during COVID-19 and beyond, a specific brief was co-developed for this vulnerable population in December 2020. Working in conjunction with disability advocates, along with academics and practitioners, this policy brief served to provide recommendations for physical activity and health in African children and adolescents with disabilities, with special reference to the COVID-19 pandemic (Naidoo et al. 2020).

Daily physical activity, mostly in the form of active play, is recommended for children and adolescents. The WHO recommends participation in moderate-to-vigorous physical activity for an average of 60 min per day. This should be composed predominantly of aerobic-type activities, integrated with some vigorous-intensity aerobic and strength activities, 3 days a week. The United Kingdom’s physical activity recommendations highlight that even 20 min per day offers health benefits, especially for children and adolescents with disabilities (Smith et al. 2022). Given the growing recognition of the importance of reducing sedentary behaviour, it has also been recommended that recreational screen time be restricted to not more than 120 min each day. Depending on the functional capabilities of children and adolescents with disabilities, supervision from a health professional may be required for the completion of certain regimens.

Physical activity policy briefs are not uncommon. However, as a direct result of the COVID-19 pandemic, a gap exists on policy guiding physical activity participation and recommendations during COVID-19, country-, culturally- and context-specific. The policy brief was to be used as a tool to present research and recommendations to non-specialised audiences.

## Policy brief development

A policy brief is a stand-alone, practical document focussing on a single topic, using an active voice, presented in clear and simple language, and providing little or no room for misinterpretation, as the target audience may not be familiar with the subject under discussion (Lund 2010; McIvor 2018). The recommendations formed for a policy brief are based on research and evaluation relevant to the setting and context to which they are to be applied (Bull et al. 2020). The most effective policy brief will present a clearly defined problem, backed up by clear evidence, and will offer realistic and cost-effective strategies to address the problem (Lund 2010). Perhaps the most important step in this process is the dissemination of the policy brief to its intended audience, through various social media platforms, the internet and/or conferences and workshops (Jones & Walsh 2008).

A policy brief is equally important to both policymakers and the target audience. It encourages conceptual use of research to influence policies by redirecting discussions to important issues concerning the public, often raising an overlooked issue for policy consideration (McIvor 2018). Policy briefs have the potential to change attitudes, social norms and behaviours, influencing both the public and government officials (Jones & Walsh 2008). Policymakers are provided with information about the problem to help them make informed decisions, to develop policies that reflect the needs of the people and to ensure service delivery. Similarly, through this process, the target audience is well informed (Sajedinejad et al. 2021). It ensures that social movements are supported and that the target audiences’ voices are heard, as they participate in decision making and exercise their rights (McIvor 2018).

This policy brief primarily focused on guiding decision makers at provincial or state, district and local levels in the development and subsequent implementation of policies and practices that promote physical activity for children and adolescents with disabilities, by creating an equitable, healthy and safe, home, school and community environment.

## The development process

The present policy brief is the third in a series of four: (1) African Physical Activity Network (AFPAN) and Academic Consortium, Policy Brief: Physical Activity for Health in Africa: Guidance for During and Beyond the COVID-19 Pandemic for the General Public, September 2020; (2) Naidoo R, Chetty V, Draper C, et al. Policy Brief: Physical Activity for Health in children and adolescents in Africa: COVID and beyond-Home, School and Communities, 16 September 2020; (3) Naidoo R, Chetty V, Smith B, et al. Policy Brief: Physical activity and health in Africa for children and adolescents with disabilities: COVID-19 and beyond-Home, School and Communities, December 2020) and (4) Christie CJ, Naidoo R, Shung-King M, Van Gent M. et al. Policy Brief: Organised school sport in South Africa for children and adolescents: COVID-19 and beyond, March 2021, which were developed by physical activity experts across the African continent.

The briefs aimed to serve as a guide to decision makers, planners and programme leaders, both during the COVID-19 pandemic and beyond. More than ever, the COVID-19 pandemic has highlighted the need to prioritise physical activity as an imperative for public health awareness in Africa, and in other lower- and middle-income countries. Hence the need for the brief was established by a group of co-leaders in the field, including researchers, healthcare experts and disability advocates across Africa, as well as partners from abroad (the United Kingdom).

A seven-step process was developed by the authors based on their previous experience when developing policy briefs one and two.

### Step 1

The academic team was formed by the co-leaders (R.N., V.C., B.S. and E.L.) of this project. Experts in this focused area were then invited to form the core writing group. An advisory group was also formed, comprising academics, researchers and stakeholders (some from the previous policy brief cohorts).

### Step 2

The core writing group (the authors) then developed a draft version of the brief, based loosely on the International Development Research Centre (IDRC) Policy Brief Toolkit (McIvor 2018). This was over a 4-week period.

### Step 3

To gain further insights into content development, co-creators, including people with disabilities; non-governmental organisations (NGOs); non-profit organisations (NPOs); athletes with disabilities; special needs teachers and parents and healthcare therapists working with children with disabilities, were involved in the co-creation of the document. This process was conducted over a 4-week period.

### Step 4

The second draft was developed and then sent to the advisory group for feedback. The advisory group was given 2-weeks to provide feedback.

### Step 5

The core writing group then developed the third draft of the brief over a 1-week period. This draft was then sent to a graphics company for formatting. This was completed within a week.

### Step 6

The third draft was circulated to the co-creators and the advisory group for final comment. Comments on the design; layout; visual appeal; understandability and importance were also requested. This was completed over a 2-week period.

### Step 7

The final version was approved by co-leaders taking into consideration the final comments. This, together with working with the graphics company was completed over a 1-week period.

The entire development process to complete the policy brief took approximately 15–16 weeks taking consideration the conceptualisation and planning times for the project.

## Policy brief summary

This policy brief can be seen in Naidoo et al. (2020) and begins with information on the benefits of physical activity for the health of children and adolescents with a disability. General guidelines on the recommended amount of physical activity are briefly presented, with the primary focus on physical activity recommendations for children and adolescents with disabilities during COVID-19, focusing on structured and unstructured lessons and play time. An infographic was developed, illustrating these recommendations, for wider dissemination.

Furthermore, we focused on the four-pillar approach: (1) Protection and safety measures; (2) Physical environment design; (3) Physical activity and physical literacy practices and (4) Physical activity sustainability, to promote healthy physical activity within healthy and safe home, school and community environments for children and adolescents with disabilities. Additional information on how to minimise the risk of injury while participating in physical activity, incorporating the use of adapted apparatus and equipment is also presented in the policy brief. Lastly, the document concludes with recommended actionable items to increase participation in safe and enjoyable physical activity, for children and adolescents with a disability.

## Challenges during the policy development process

The development of the brief was challenging during the COVID-19 lockdown. The ideal scenario would have been to meet in person. However, due to restrictions on movement and social interaction, the information was compiled through remote collaboration due to the urgency of the document. The challenge was the turnaround time to respond and solicit feedback from collaborators. The co-leaders had the task of then considering all the comments and drafting a paper that was circulated a few times for approval. It should be noted that the collaborators were challenged by the changing work environment of higher education institutions, as well as personal matters such as dealing with the pandemic themselves.

## Conclusion

The ‘Policy Brief: Physical activity and Health in Africa for Children and Adolescents with Disabilities: COVID-19 and Beyond’ offers policymakers a sound roadmap on how to address inactivity during the present pandemic. Although the brief has been distributed to both the public and private sectors, the Ministries of Sport and Recreation, Education and Health now need to use it in the development of their policies.

This policy brief must also be distributed to universities training students who will work with children with special needs and disabilities, so that this can be incorporated into their programme development. Further dissemination of the policy brief is necessary, and public forum opportunities and conferences should be used as platforms in order to present the brief.

Given that the WHO’s stance stresses the prevention of disease through behaviour modification, future collaborative studies between African universities should explore ways to track and enhance physical activity, particularly among marginalised populations with disabilities.
